# Analysis of Antigen Expression in T-Cell Acute Lymphoblastic Leukemia by Multicolor Flow Cytometry: Implications for the Detection of Measurable Residual Disease

**DOI:** 10.3390/ijms26052002

**Published:** 2025-02-25

**Authors:** Alexandra Semchenkova, Ekaterina Mikhailova, Irina Demina, Julia Roumiantseva, Alexander Karachunskiy, Galina Novichkova, Alexander Popov

**Affiliations:** Dmitry Rogachev National Medical Research Center of Pediatric Hematology, Oncology and Immunology, 1 Samory Mashela St., Moscow 117198, Russia; semalex94@mail.ru (A.S.);

**Keywords:** measurable residual disease, T-cell acute lymphoblastic leukemia, flow cytometry

## Abstract

Multicolor flow cytometry (MFC) is a key method for assessing measurable residual disease (MRD) in acute lymphoblastic leukemia (ALL). However, very few approaches were developed for MRD in T-cell ALL (T-ALL). To identify MRD markers suitable for T-ALL, we analyzed the expression of CD2, CD3, CD4, CD5, CD7, CD8, CD10, CD34, CD45, CD48, CD56, CD99, and HLA-DR in T-ALL patients at diagnosis. The median fluorescence intensities (MFIs) of surface CD3, CD4, CD5, CD7, CD8, CD45, CD48, CD99, and CD16+CD56 were also evaluated at Day 15 and the end-of-induction (EOI). The MFC data from 198 pediatric T-ALL patients were analyzed retrospectively. At diagnosis, the most common antigens were identified, and the MFI of T-lineage antigens in blasts was compared to that in T lymphocytes. At follow-up, the MFIs of the proposed MRD markers were compared to those observed at diagnosis. The most common T-ALL antigens were CD7 (100.0%), intracellular CD3 (100.0%), CD45 (98.5%), and CD5 (90.9%). The MFIs of T-lineage antigens in blasts differed significantly from those in T lymphocytes. By the EOI, a substantial modulation of sCD3, CD4, CD5, CD7, CD8, and CD45 was observed. CD48 and CD99 were the most stable markers. The proposed MRD markers (sCD3, CD4, CD5, CD7, CD8, CD45, CD48, CD99, CD16+CD56) enabled MFC-MRD monitoring in virtually all T-ALL patients.

## 1. Introduction

Multicolor flow cytometry (MFC) is a cornerstone method for acute lymphoblastic leukemia (ALL) diagnosis [1,2]. MFC provides a comprehensive analysis of surface and intracellular antigens in leukemic cells, which allows leukemia lineage assignment, risk stratification, and the identification of potential therapeutic targets [3,4]. Leukemic immunophenotypes established at diagnosis also form the basis for monitoring measurable residual disease (MRD) during therapy. With well-designed antibody panels and an increasing number of analyzed cells, MFC has become a powerful tool for detecting very low levels of residual blasts [2,5]. MRD status has major clinical applications beyond evaluating therapeutic efficacy. The kinetics of leukemia clearance provide valuable prognostic information and a foundation for clinical decision-making in the treatment of ALL arising from B-cell precursors (BCP-ALL) and T cells (T-ALL) [5,6,7].

Different groups of experts in the field have proposed MFC-based approaches for MRD detection [8,9,10,11]. Most developed guidelines have focused on MRD analysis for BCP-ALL. The algorithms described provide highly sensitive and well-harmonized detection of residual leukemia regardless of which therapy is applied. On the other hand, descriptions of MFC-MRD assessment in T-ALL patients are rare in the literature. To date, only a few MRD-targeted antibody combinations and analysis strategies have been reported [7,12,13,14,15,16]. The search for a reliable combination of markers to distinguish leukemic T lymphoblasts from mature T and NK cells is still ongoing. It is equally important to eliminate the use of multiple tubes for MRD assessment in favor of a single MRD tube. Various precursor markers, such as CD99, terminal deoxynucleotidyl transferase (TdT), and CD34, as well as conventional lymphocytic antigens, have been introduced at different times as potential targets for MRD testing [13,15,17,18]. However, a universal gating approach or antibody panel has not yet been developed.

In this study, we analyzed the expression levels of multiple T-cell-associated markers (CD2, surface (s) and intracellular (i) CD3, CD4, CD5, CD7, and CD8) and others (CD10, CD34, CD45, CD48, CD99, and HLA-DR) in the bone marrow (BM) at diagnosis and during follow-up in pediatric patients with T-ALL.

## 2. Results

### 2.1. Diagnostic Immunophenotyping

Among the 198 T-ALL patients included in this study, 27 had early T-cell precursor ALL (ETP-ALL) (13.6%) and were classified as either TI (*n* = 9) or TII (*n* = 18) according to the criteria of the European Group for the Immunological Characterization of Leukemias (EGIL). The remaining patients had either non-ETP TII (*n* = 51, 25.8%), TIII (*n* = 109, 55.1%), or TIV (*n* = 11, 5.6%) ALL subtypes. The most frequently expressed antigens of other hematopoietic lineages were CD117 (*n* = 54, 27.3%), CD33 (*n* = 39, 19.7%), and iCD79a (*n* = 32, 16.2%). CD56 was expressed in 18 cases (9.1%). The diagnostic results are summarized in Table 1.

### 2.2. Analysis of Antigen Expression on Leukemic T Lymphoblasts at Diagnosis

**Figure 1:**
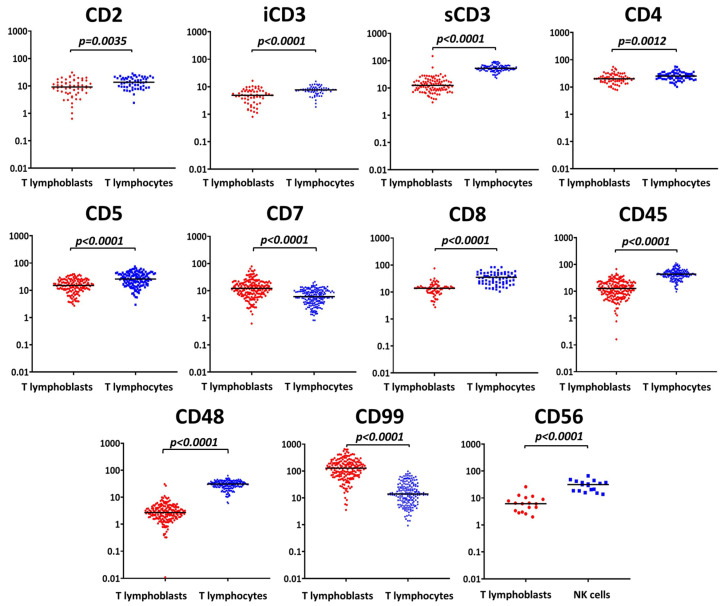
Comparison of MFI values obtained from T lymphoblasts and mature T lymphocytes or NK cells. Y-axis, MFI values (each data point represents an individual sample).

All the information obtained about antigen expression is summarized in Table 2 and Table 3, and Figure 1.

#### 2.2.1. CD10, CD34 and HLA-DR

Among the immaturity-associated markers used in the diagnostic panel (CD10, CD34, HLA-DR), CD34 was the most common (*n* = 116, 58.6%) and expressed heterogeneously (*n* = 85, 42.9%) (Table 2). The mean percentage of CD34-positive cells was 67%. CD10 was positive in 77 samples (39.3%; mean expression 77%), while HLA-DR-positive T lymphoblasts were present in a minority of patients (*n* = 39, 19.8%; mean expression 62%), mainly in those with ETP-ALL (*n* = 21).

#### 2.2.2. CD2

At diagnosis, CD2 expression was detected on leukemic blasts from 153 patients (77.3%) (Table 2). Most patients (*n* = 126, 63.6%) demonstrated high levels of this antigen, and the mean expression among all CD2-positive leukemic populations was 91%. Of the patients with CD2-negative blasts, 14 were diagnosed with ETP-ALL (TI, *n* = 9; TII, *n* = 5), 16 had non-ETP TII ALL, and 11 and 4 patients had TIII and TIV, respectively. The median fluorescence intensity (MFI) of CD2-positive lymphoblasts was significantly lower than that of mature T lymphocytes (median CD2 MFI quotient, MFIQ, 0.89, range 0.03–2.07, *p* = 0.0035) (Table 3, Figure 1).

#### 2.2.3. CD3 and CD5

As expected, iCD3 was expressed in leukemic T lymphoblasts in all cases, with a mean positivity of 65% (Table 2). The median iCD3 MFI of T lymphoblasts differed by 0.71 times from that of T lymphocytes (range 0.11–1.84, *p* < 0.0001) (Table 3, Figure 1). The surface expression of CD3 (sCD3) was negative in 77 out of 198 patients (38.9%), and the majority of them (*n* = 39) were diagnosed with TII (non-ETP-ALL, *n* = 25; ETP-ALL, *n* = 14). In this study, sCD3 was highly expressed in 32 patients (16.2%), mostly those with TIII (*n* = 20) or TIV (*n* = 7) ALL. Heterogeneous expression was found in 89 cases (44.9%). The mean sCD3 expression in all samples with sCD3-positive blasts (*n* = 121) was 58%. T lymphocytes had significantly greater sCD3 MFI values than did T lymphoblasts (median sCD3 MFIQ 0.23, range 0.04–1.80, *p* < 0.0001) (Table 3, Figure 1).

Eighteen patients (9.1%) had CD5-negative leukemic populations and were mainly diagnosed with ETP-ALL (*n* = 16). In the remaining 180 patients, CD5 expression was heterogeneous in 28 patients (14.1%), while 152 patients (76.8%) had high levels of this marker (Table 2). The mean CD5 expression was 93%. CD5 MFI values of T lymphoblasts were also significantly lower (median CD5 MFIQ 0.64, range 0.07–3.52, *p* < 0.0001) (Table 3, Figure 1).

**Figure 2:**
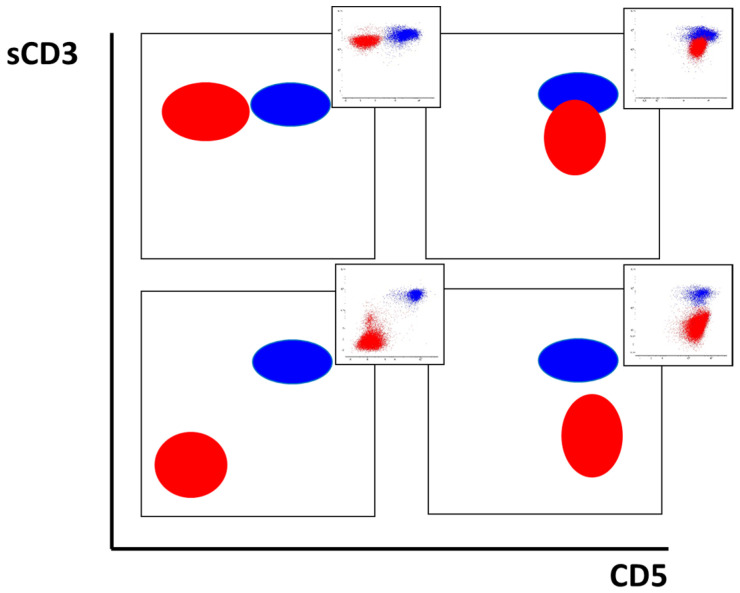
Different variants of surface CD3 and CD5 expression in T-ALL patients. Leukemic cells are shown in red and mature T cells are shown in blue. Exemplary cases are presented in the top right corner of each plot.

Dot plots illustrating different patterns of mutual expression of sCD3 and CD5 are shown in Figure 2.

#### 2.2.4. CD4 and CD8

Analysis of the mutual expression of CD4 and CD8 revealed a significant proportion of patients with blasts negative for both markers (*n* = 90, 45.5%). Sixty patients (30.3%) had blasts positive for both CD4 and CD8; 28 (14.1%) and 20 patients (10.1%) demonstrated CD4(−)CD8(+) and CD4(+)CD8(−) phenotypes, respectively (Table 1).

The mean CD4 expression was 59% (Table 2). Mature CD4-positive T lymphocytes had significantly greater CD4 MFI values (median CD4 MFIQ 0.82, range 0.28–2.31, *p* = 0.0012) (Table 3, Figure 1).

The mean CD8 expression among all CD8-positive leukemic populations was 70% (Table 2), and their CD8 MFI values were significantly lower than those of CD8-positive T lymphocytes (median CD8 MFIQ 0.40, range 0.10–1.85, *p* < 0.0001) (Table 3, Figure 1).

**Figure 3:**
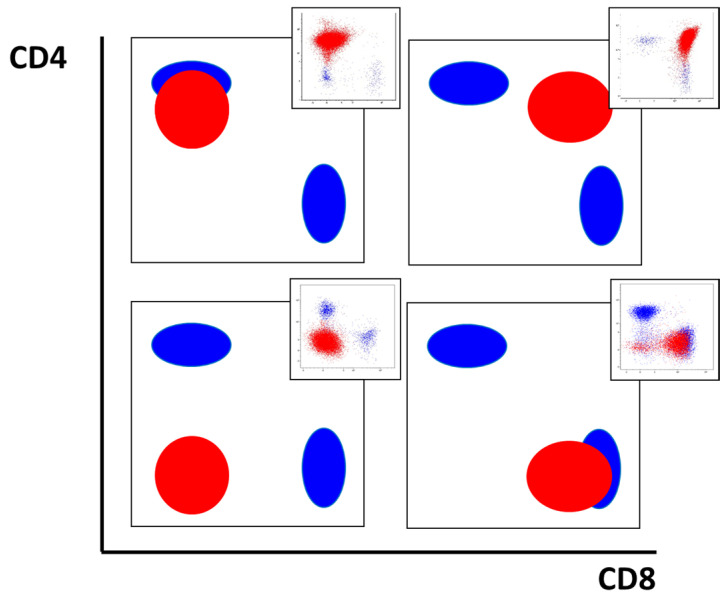
Different variants of CD4 and CD8 expression in T-ALL patients. Leukemic cells are shown in red and mature T cells are shown in blue. Exemplary cases are presented in the top right corner of each plot.

Figure 3 shows typical patterns of CD4 and CD8 expression.

#### 2.2.5. CD7

In all patients, leukemic populations were positive for CD7. The mean CD7 expression reported was 98% (Table 2). Most patients (*n* = 190, 96.0%) had ≥90% blasts positive for CD7. The expression of this marker was significantly greater on T lymphoblasts than on normal T lymphocytes based on a median CD7 MFIQ of 2.13 (range 0.22–19.01; *p* < 0.0001) (Table 3, Figure 1).

#### 2.2.6. CD45

**Figure 4:**
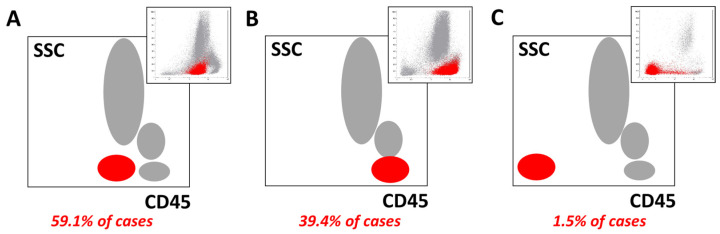
CD45 expression variants observed in leukemic T lymphoblasts and their frequency among T-ALL patients. Most patients demonstrated either medium (**A**) or strong (**B**) positivity for CD45. CD45 negativity was the least common (**C**). Leukemic cells are shown in red and other nucleated cells are shown in gray. Exemplary cases are presented in the top right corner of each plot.

In 117 out of 198 cases (59.1%), ≥50% of T lymphoblasts expressed CD45 at the intensity level of mature granulocytes (Figure 4A). In 78 samples (39.4%), leukemic blasts had the same intensity of CD45 expression, as did mature lymphocytes (Figure 4B). Only 3 out of 198 patients (1.5%) had leukemic populations that were completely negative for CD45 (TIII, *n* = 2; TII, *n* = 1) (Figure 4C). Overall, the CD45 MFI values of mature T lymphocytes were greater than those of leukemic blasts (median CD45 MFIQ 0.33, range 0.00–1.66, *p* < 0.0001) (Table 3, Figure 1). Examples of CD45 expression are presented in Figure 4.

#### 2.2.7. CD48 and CD99

**Figure 5:**
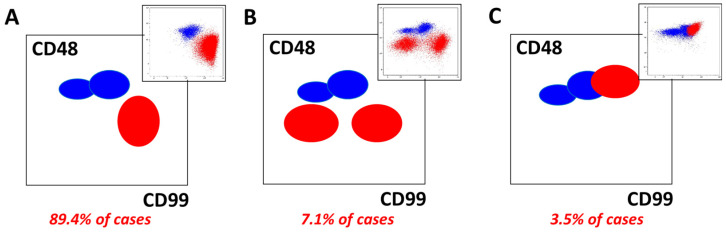
Different patterns of CD48 and CD99 expression observed on leukemic T lymphoblasts and their frequency among T-ALL patients. Most patients had leukemic populations that were negative for CD48 and homogeneously positive for CD99 (**A**). T lymphoblasts and T lymphocytes could be divided on such dot plots. In some cases, leukemic populations were divided into two subpopulations with different CD99 expression levels (**B**). In a minority of cases, ≥50% of leukemic cells had the same level of CD48 and CD99 expression and could not be distinguished from T lymphocytes on such dot plots (**C**). Leukemic cells are shown in red, and mature T cells are shown in blue. Exemplary cases are presented in the top right corner of each plot.

The CD48 MFI of mature T lymphocytes was significantly greater than that of leukemic blasts (median CD48 MFIQ 0.10, range 0.00–1.21, *p* < 0.0001) (Table 3, Figure 1). The opposite pattern was observed for the CD99 MFI signals. The median CD99 MFI of T lymphoblasts was 10.13 times greater (range 0.34–47.05) than that of mature T lymphocytes (Table 3, Figure 1). In most samples (*n* = 177, 89.4%), a visible difference was observed between T lymphocytes and T lymphoblasts with respect to CD48 and CD99 expression (Figure 5A). Additionally, in 14 samples (7.1%), there were two leukemic subpopulations with different levels of CD99 expression (Figure 5B). Typically, one subpopulation had the same CD99 MFI as that of mature T cells, while the second had a median CD99 MFI of 46.77 times greater (range 9.49–l05.70). In seven patients (3.5%), the expression of CD48 and CD99 on T lymphoblasts was similar to that on T lymphocytes (Figure 5C). Examples of the mutual expression of CD48 and CD99 are illustrated in Figure 5.

#### 2.2.8. CD56

Among the CD56-positive leukemic populations (*n* = 18, 9.1%), this marker was mostly heterogeneously expressed (*n* = 14) (Table 2), and only four samples demonstrated high levels of expression (ETP-ALL TI, *n* = 1; non-ETP ALL TII, *n* = 2; TIV, *n* = 1). The MFI signals from CD56-positive fractions of leukemic populations were significantly dimmer than those of normal NK cells (median CD56 MFIQ 0.25, range 0.05–0.76, *p* < 0.0001) (Table 3, Figure 1).

### 2.3. Immunophenotypic Features of ETP-ALL at Diagnosis

This study included 27 patients with ETP-ALL. Of them, 26 presented with de novo disease, and one was studied for relapse confirmation. In all patients, leukemic blasts expressed CD7 (mean expression: 95%, range: 52–100%), iCD3 (mean: 46%, range: 17–100%), and CD45 (mean: 99%, range: 86–100%). Among the T-lineage antigens, CD2 was detected in 13 patients (48.1%) (mean expression: 92%, range: 29–100%), and CD5 was detected in 11 ETP-ALL patients (40.7%), with a mean expression of 48% (range: 28–68%). Surface CD3 was the least expressed T-lineage marker (*n* = 5, 18.5%), with a mean positivity of 31% (range: 21–52%). Most ETP-ALL cases (*n* = 26, 96.3%) had blasts negative for CD4 and CD8, with only one patient (3.7%) having CD4-positive blasts. Among the immaturity-associated markers, CD34 and HLA-DR were the most frequently expressed. CD34 was detected in 24 patients (88.9%), with a mean expression of 90% (range: 50–100%), while HLA-DR was detected in 21 cases (77.8%). In three patients (11.1%), leukemic blasts were positive for CD10 (mean expression: 100%). In most patients (*n* = 26, 96.3%), the expression of CD48 and CD99 on T lymphoblasts differed from that on mature T cells, allowing differentiation between these two populations.

At diagnosis, three ETP-ALL patients (11.1%) had additional leukemic populations of another lineage. Only two of these patients were subsequently investigated for MRD, and no evidence of non-T-cell leukemic blasts was found.

Among the 26 newly diagnosed patients, only 20 (76.9%) were investigated for MRD. At Day 15, 13 of 14 studied patients (50.0% of all newly diagnosed ETP-ALL patients) were MRD-positive (median MRD: 11.925%, range: 0.008–69.600%). At the end of induction therapy (EOI), 6 of 15 studied patients (23.1% of all newly diagnosed ETP-ALL patients) were MRD-positive (median MRD: 0.045%, range: 0.025–0.531%). Only ten newly diagnosed ETP-ALL patients (38.5%) were studied at both MRD timepoints. Two patients switched to myeloid leukemia at Day 15, and both completely lost their initial T lymphoblasts. However, with our T-cell MRD-oriented tube, it was possible to detect myeloid leukemic cells in both cases. One patient lost CD7 expression completely, while the other retained partial CD7 expression.

### 2.4. Immunophenotypic Features of Mature T-ALL at Diagnosis

Overall, 25 patients (12.6%) demonstrated features of a mature T-lineage immunophenotype at diagnosis, e.g., the presence of T-cell receptor (TCR) chains. Most had blasts with a TIII immunophenotype (*n* = 14) and TCRγδ expression (*n* = 8). Eleven patients were diagnosed with TIV ALL, with TCRγδ expression being the most common (*n* = 9). In all patients with mature immunophenotypes, T lymphoblasts were positive for CD7 (mean expression: 100%), iCD3 (mean: 82%, range: 14–100%), CD3 (mean: 88%, range: 64–100%), and CD45 (mean: 98%, range: 76–100%). CD2 was detected in 18 patients (72.0%), with a mean expression of 82% (range: 33–100%), while CD5 was detected in 24 patients (96.0%) (mean: 94%, range: 20–100%). Thirteen patients (52.0%) had blasts positive for CD8 (mean: 72%, range: 0–100%), and 12 patients (48.0%) had CD4 expression (mean: 64%, range: 22–100%). Among the immaturity-associated markers, CD10 and CD34 were the most frequently expressed (CD10, *n* = 11, 44.0%; CD34, *n* = 8, 32.0%). HLA-DR was positive in two patients (8.0%). In most patients (*n* = 21, 84.0%), T lymphoblasts and T lymphocytes could be accurately distinguished by the expression of CD48 and CD99.

Among the 25 patients with a mature T-lineage immunophenotype, 22 (88.0%) were newly diagnosed. At Day 15, 17 patients were studied for MRD, and 16 of them were MRD-positive (median MRD: 0.984%, range: 0.001–57.061%). At EOI, 16 patients were studied, 5 of whom were MRD-positive (median: 0.505%, range: 0.104–0.864%).

### 2.5. Results of MFC-MRD Testing at Follow-Up and Comparison of Antigen Expression

MRD investigation at Day 15 was performed for 114 patients (61.3%) of the newly diagnosed patients with T-ALL (*n* = 186). In three patients, proper results could not be obtained because of poor sample quality, and 88 out of 114 patients (77.2%) were MFC-MRD-positive (median MRD: 1.540%, range: 0.001–90.960%). Among them, two patients underwent lineage switching from ETP-ALL to myeloid leukemia, and were therefore excluded from analysis. MFC-MRD results at EOI were available for 133 patients (71.5%), and MRD-positive results were obtained for 41 samples (30.8%). The median percentage of residual blasts at EOI was 0.070 (range: 0.001–2.490%).

**Figure 6:**
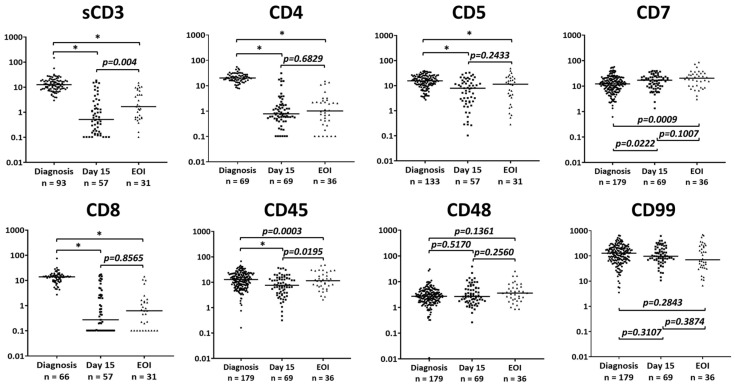
Changes in the MFIs of sCD3, CD4, CD5, CD7, CD8, CD45, CD48, and CD99 observed in leukemic blasts during induction therapy in patients with T-ALL. The levels of statistical significance are given above the respective intervals investigated. * *p*-value <0.0001. Y-axis, MFI values (each data point represents an individual sample).

The MFI values of antigens included in our MRD-oriented panel (sCD3, CD4, CD5, CD7, CD8, CD45, CD48, and CD99) were obtained from MRD samples stained with identical antibody-fluorochrome combinations and acquired with identical cytometer settings. These were then compared with the relevant MFI values observed in identically processed samples of newly diagnosed patients. The results are shown in Figure 6 and Table A1. By EOI, we observed a significant decrease in MFI of most antigens: sCD3 (*p* < 0.0001), CD4 (*p* < 0.0001), CD5 (*p* < 0.0001), CD8 (*p* < 0.0001), and CD45 (*p* = 0.0003). The MFI of CD7, in contrast, had increased by EOI (*p* = 0.0009). Modulation of these markers was already observed at Day 15. The MFI of sCD3 and CD45 continued to decrease by EOI (*p* = 0.004 and *p* = 0.0195 respectively, Day 15 vs. EOI). However, no significant difference in the MFI of CD4 (*p* = 0.6829), CD5 (*p* = 0.2433), or CD8 (*p* = 0.8565) was detected between the Day 15 and EOI timepoints. The most stable expression was detected for CD48 (*p* = 0.1361, diagnosis vs. EOI) and CD99 (*p* = 0.2843, diagnosis vs. EOI).

## 3. Discussion

Similarly to that in BCP-ALL patients, MRD in pediatric T-ALL patients determined using either MFC or molecular techniques has strong and independent prognostic value [7,14,19,20,21,22]. At the same time, MFC-based MRD monitoring is less developed for the detection of residual T lymphoblasts than for the detection of residual leukemia in BCP-ALL patients. The selection of markers for MRD studies in these two types of leukemia is based on different approaches [23]. In T-ALL, the basic principle is to identify cells with immunophenotypic features of immature T lymphocytes [2,24]. This is due to the fact that nonmalignant thymocytes should not be detected in the BM, as they normally develop in the thymus. The earliest thymocytes express CD2, iCD3, and CD7, and thus these antigens are typically included in the MRD panel [25]. The next stage of development is associated with the expression of CD1a and double positivity for CD4 and CD8 [25]. Historically, MRD studies were based on the detection of TdT-positive T cells [12,18] or the simultaneous use of common T-lineage antigens and immaturity-associated markers (CD99, CD34, CD1a, and CD10) [24].

As we do not use TdT in diagnostic and follow-up workflows [26], it was not studied in the context of this research. The other immature antigens investigated in our study are typically heterogeneously expressed, which severely limits their applicability for MFC-MRD investigations. Indeed, in the case of partial positivity, it is difficult to gate the entire residual leukemic population using such heterogeneously expressed antigens. They become applicable only for further characterization of previously gated cells, as they may indicate an immunophenotypic aberration. At the same time, expanding the antibody panel with rarely applicable antigens makes MFC-MRD monitoring even less harmonized and reproducible. Additionally, early antigens are often downregulated or even lost during the initial stages of ALL-directed chemotherapy [17,27,28]. Although the use of early progenitor antigens is still a popular approach for MFC-MRD monitoring in the T-ALL [2,13,14,15], it seems futile because of its very limited usefulness and ability to expand and customize antibody panels.

The use of common T-lineage antigens (CD7, CD2, CD3, CD5, CD4, and CD8) is based mainly on their aberrant expression compared to that of normal mature T lymphocytes [24]. All the mentioned antigens typically display bright, homogeneous positivity on normal T cells. Therefore, decreased or increased CD7, CD2, CD5, and CD3 expression is the main immunophenotypic aberration, although for the CD4/CD8 pair, the absence of restriction to one of these markers can also be considered a leukemic-associated immunophenotype. Leukemic cells were homogeneously positive for CD7 and CD5 in almost all studied samples, while CD2 positivity was less common. The most mature antigen, surface CD3, was expressed in a small number of cases and in a very heterogeneous way. All the studied antigens displayed differences in expression between leukemic blasts and normal T cells. On the other hand, the aberrant expression of each antigen was not found in all cases, or for a substantial proportion of patients, the expression did not differ enough to distinguish between residual leukemic cells and their normal counterparts. Moreover, low numbers of cells with decreased or increased expression of common T-lymphoid antigens can be found in normal BM [29], thus mimicking MRD in T-ALL. CD4/CD8-negative (referred to as ‘double negative’) and CD4/CD8-positive (referred to as ‘double positive’) normal cells have been found in three out of four children with T-ALL [29]. Finally, all of the abovementioned markers displayed treatment-related changes in expression. Taken together, these findings indicate the necessity of using a set of T-lineage antigens, as it is impossible to rely on a single antigen or even a pair of antigens displaying typical normal and leukemic patterns on a dot plot. To avoid overloading the panel, we suggest omitting CD2, as it was the rarest early T-lineage marker (compared to CD7 and CD5), and the difference in expression between normal and leukemic T cells was less visible. We also prefer not to use iCD3. Evaluation of intracellular expression requires an additional permeabilization step during sample preparation. This leads to more significant cell loss, which is crucial for MRD investigations. Moreover, such complicated preanalytical steps may result in less than total intracellular CD3 expression. Considering that this antigen is mainly included in antibody combinations for precise T-lineage compartment gating, its usefulness seems doubtful. Among all CD7-positive cells, NK cells can be excluded by CD16 and CD56 positivity, as CD56 was found to be very rarely expressed in patients with T-ALL. This finding also agrees well with published data [30]. In our study, two antigens, CD99 and CD48, showed great applicability for MFC-MRD monitoring in T-ALL. Although CD99 is already a more or less traditional marker for leukemic T lymphoblasts [17], CD48 is less known for this application [31]. Recently, F. Kowarsch et al. published the results of their study based on the applicability of computational estimation of different antigens for MFC-MRD detection in T-ALL [16]. CD48 and CD99 were also described as the most informative markers in the multicenter setting; either when used separately from each other or in combination. Our data are in line with published results and establish these antigens as necessary components of the MFC-MRD evaluation pipeline for pediatric patients with T-ALL.

In addition to T-lineage antigens, CD48 and CD99 should also be used as necessary antibodies. Finally, although a decrease in CD45 expression is not as obvious a leukemia-associated aberration in T-ALL as it is in BCP-ALL [32], this antigen is useful for a substantial number of cases. As a result, we suggest the use of a 9-color (10 antibodies) panel (CD7, CD5, sCD3, CD4, CD8, CD45, CD48, CD99, and CD16+CD56) augmented with SYTO41 as a nucleic acid dye as the most appropriate antibody set for MFC-MRD monitoring in T-ALL. In this approach, CD7 is applicable for the primary gating of the T/NK cell compartment and CD16+CD56 is suggested for the exclusion of NK cells from the analysis, while the remaining antigens could be used for the precise identification of blasts concerning both the initial immunophenotype and the differences between normal and leukemic cells. Compared to other published approaches, we used only one antibody combination, which does not include immaturity-associated markers and avoids intracellular staining. These peculiarities allow greater cell input within a single tube without additional cell loss due to permeabilization. Moreover, such a panel requires the use of 10-color cytometry, which can be appropriately harmonized or even standardized in a modern setting. Regardless of the antigen positivity thresholds used for initial diagnosis, this approach reliably identifies leukemic blasts, as it is primarily based on differences in antigen expression levels between T lymphoblasts and mature T cells. This ensures a robust and consistent detection of leukemic populations, even in the presence of variability in diagnostic criteria.

This combination of antibodies is highly suitable for MFC-MRD monitoring in virtually all T-ALL patients, as the expression of the included T-lineage antigens is typically characteristic of leukemic T-lymphoblasts. Furthermore, the combination of CD48 and CD99 alone provides a clear distinction between mature T lymphocytes and leukemic cells in the overwhelming majority of cases (96.5% in the present cohort). However, the application of this approach may theoretically be limited in situations where the immunophenotype of T lymphoblasts completely overlaps with that of mature T cells, particularly in cases exhibiting high CD48 expression and reduced CD99 levels. Nevertheless, such cases are exceptionally rare.

## 4. Materials and Methods

We performed a retrospective review of the records of patients diagnosed with T-ALL at our institution. BM aspirates from 2448 patients were analyzed to confirm a diagnosis of acute leukemia between June 2021 and December 2022. A total of 198 patients (median age 6.2 years; range 4 months—17.4 years) were diagnosed with de novo (*n* = 186) or relapsed (*n* = 12) T-ALL. Three patients were under one year of age. The ALL was diagnosed using morphological, cytochemical, immunophenotypic, and cytogenetic studies, as described elsewhere [33]. All newly diagnosed patients were enrolled in either ALL Moscow-Berlin (MB)-2015 (*n* = 177) [34,35] or Berlin-Frankfurt-Münster (BFM)-based (*n* = 9) [36] treatment protocol. Relapsed patients (*n* = 12) were treated with the ALL-Rez 2016 protocol [37].

### 4.1. Immunophenotyping at Diagnosis

**Figure 7:**
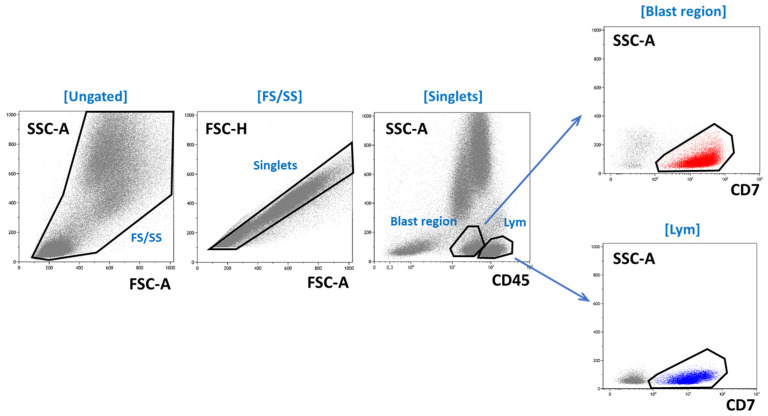
The backbone gating of T lymphoblasts and T lymphocytes at diagnosis. Leukemic cells are shown in red, mature T cells in blue, and other BM cells in gray.

At diagnosis, BM aspirates of the T-ALL patients (*n* = 198) were stained with a four-tube multicolor panel of antibodies (Table 4) and a set of MRD-oriented antibodies with addition of the fluorescent nucleic acid dye SYTO41 (Thermo Fisher Scientific, Waltham, MA, USA) (Table 5). Sample preparation was performed using the standard stain/lyse/wash method using BD FACS Lyse buffer (Becton Dickinson, BD, San Jose, CA, USA) and phosphate-buffered saline (BD Cell Wash, BD). Intracellular staining was performed using a BD IntraSure kit (BD) for fixation and permeabilization. The general approach to immunophenotyping was based on the guidelines of the Russian-Belarusian multicenter group for childhood ALL [26]. The backbone gating of leukemic blasts at diagnosis is shown in Figure 7. After exclusion of doublets, the blast region was delineated on the CD45/side scatter (SSC) dot plot and then purified by CD7 expression as appropriate [38]. The T-ALL subtypes were established according to the EGIL system [39], with subsequent modifications [26,38]. ETP-ALL was diagnosed according to well-known criteria [40] and reported in addition to the TI or TII EGIL subtypes.

The stained samples were acquired on a three-laser Navios cytometer (Beckman Coulter, BC, Marseilles, France). The EuroFlow guidelines for machine performance monitoring were used. Flow-Check Pro Fluorospheres (BC) were used for daily cytometer optimization. At least 20,000 leukemic blasts were collected per tube. The data were then analyzed using Kaluza Analysis 2.1 software (BC). The cutoff levels for antigen positivity were established at 20% for surface antigens and 10% for intracellular antigens [26,39].

#### Evaluation of Antigen Expression at Diagnosis

To identify markers potentially useful for MFC-based MRD monitoring, we analyzed the following antigens included in our staining panel: CD2, iCD3, sCD3, CD4, CD5, CD7, CD8, CD10, CD34, CD45, CD48, CD56, CD99, and HLA-DR. In this regard, two characteristics of leukemic blasts were assessed:

- Expression (%) of CD2, iCD3, sCD3, CD4, CD5, CD7, CD8, CD10, CD34, CD56, and HLA-DR;
- MFIs of CD2, iCD3, sCD3, CD4, CD5, CD7, CD8, CD56, CD45, CD48, and CD99.

The antigen expression was reported as follows: (1) high (if ≥90% of cells were antigen-positive); (2) heterogeneous (if ≥20% (10% for intracellular markers) and <90% of cells were antigen-positive); and (3) negative (if <20% (10% for intracellular markers) of cells were antigen-positive). In each sample, normal lymphocytes were used as an internal control.

The MFI values of CD2, iCD3, sCD3, CD4, CD5, CD8 were calculated for antigen-positive fractions of the leukemic populations and compared with the corresponding MFI values of mature T lymphocytes. CD56 MFI values were also calculated for CD56-positive blasts and compared with the CD56 MFI values of NK cells. This methodology was employed to ascertain the degree of difference in the MFI between antigen-positive blasts and T lymphocytes/NK cells, as this is a crucial factor in differentiating between blasts and normal cells when examining MRD. The MFI values of CD7, CD45, CD48, and CD99 were calculated for the entire leukemic population and compared with the corresponding MFI values of T lymphocytes. MFIQ values were obtained by dividing the MFI values of leukemic populations by the MFI values of their normal counterparts (T lymphocytes or NK cells) in individual samples.

The expression of sCD3, CD4, CD5, CD7, CD8, and CD45 (both % and MFI values) was assessed in the MRD-oriented tube.

### 4.2. Monitoring of Measurable Residual Disease

The study of antigen expression and shifts during therapy was limited to patients with de novo T-ALL (*n* = 186) who received conventional induction chemotherapy. Patients with relapsed T-ALL (*n* = 12) were excluded from the analysis. Regardless of the therapy protocol, MRD assessment was performed on BM samples in the middle of induction therapy (Day 15) and at EOI (Day 36 or 33 in the different protocols applied).

For patients with the TI, TII or TIII (TCRαβ- and TCRγδ-negative) ALL subtypes, the MRD-oriented tube from the diagnostic panel (Table 5) was also used for the MRD studies. For patients with TCRαβ/TCRγδ-positive T-ALL, the MRD study was performed using a different antibody combination. Therefore, these patients were excluded from the analysis. Sample preparation was performed using the standard stain/lyse/wash method using BD FACS Lyse buffer (BD) and phosphate-buffered saline (BD). Cell debris and nonnucleated cells were excluded from analysis by using SYTO41 dye. Samples were also acquired on a Navios cytometer (BC). Flow-Check Pro Fluorospheres (BC) were used for daily cytometer optimization. In each case, at least 300,000 SYTO41-positive events were collected. The low limit of detection was established at 10 clustered dots with specific immunophenotypes and lymphoid light-scatter parameters. The proportion of the leukemic cell population was presented as the percentage of SYTO41-positive cells.

In each sample, the gating of residual blasts began with the removal of doublets and debris on several consecutive dot plots. Then, all T-lineage cells were gated based on CD7 expression based on appropriate light-scatter characteristics. A multiparameter analysis was then applied to identify abnormal T lymphoblasts. In each case, the search was based primarily on the exclusion of mature T lymphocytes and NK cells but with respect to the leukemia-associated immunophenotypes observed at diagnosis. For every sample examined for MRD, the gating strategy was composed individually.

**Figure 8:**
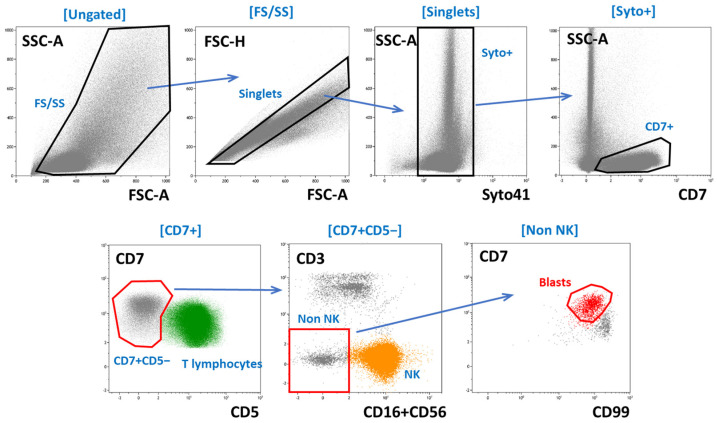
An exemplary gating of MRD cells in a T-ALL patient. At diagnosis, blasts were positive for CD7 and CD45 and negative for sCD3, CD5, and CD56. The top row depicts the sequential exclusion of doublets and non-nucleated cells. The bottom row shows the sequential exclusion of normal T lymphocytes (green) and NK cells (orange) and the gating of residual blasts (red). Other BM cells are shown in gray.

An exemplary case of the MRD study is shown in Figure 8.

#### Evaluation of Antigen Expression at Follow-Up

We evaluated the MFI values of sCD3, CD4, CD5, CD7, CD8, CD45, CD48, and CD99 in MRD-positive samples at Day 15 and EOI and compared with the corresponding MFI values observed at diagnosis. In addition, MFI levels were compared between Day 15 and EOI if the leukemic population persisted. All comparisons were performed for samples stained with the same antibody-fluorochrome combinations and acquired with the same cytometer settings.

### 4.3. Statistical Analysis

Descriptive statistics (mean, median, range) were applied to all numerical data at diagnosis and follow-up. Mean was used to describe the proportions of leukemic blasts and antigen positivity; median was used to describe the age of patients, MFI/MFIQ values, and % MRD. The normality of the MFI datasets of T lymphoblasts and T lymphocytes was tested using the Shapiro–Wilk test. The Wilcoxon matched-pairs signed rank test was used to compare the median MFI values of T lymphoblasts and T lymphocytes. The Mann–Whitney test was used to compare the median MFI values of antigens between two time points. The Kruskal–Wallis test was applied to compare the MFI values obtained at three time points (diagnosis, Day 15, and EOI). A *p*-value of 0.05 or lower was considered statistically significant. All the statistical tests and their visualization were performed using GraphPad Prism 8.0.1 software.

## 5. Conclusions

In our study, we evaluated the initial expression and treatment-related changes in antigens proposed by different groups for MFC-MRD detection in T-ALL patients. Our findings enabled us to exclude immature antigens, intracellular markers, and several T-lineage antigens to develop a fully reproducible approach that allows MFC-MRD monitoring in nearly all T-ALL patients. The proposed antibody panel can be used for highly harmonized routine laboratory practice, and the results obtained could be used for patient stratification.

## Figures and Tables

**Table 1:** Results of the diagnostic immunophenotyping of T-ALL patients.

Characteristic	N (%)
Total patients	198 (100.0)
Gender	
Male	151 (76.3)
Female	47 (23.7)
Median age (range, y)	6.2 (0.3–17.4)
≤1 year	3 (1.5)
Mean % blasts (range, %)	71 (9–98)
ETP-ALL	27 (13.6)
TI	9 (4.5)
TII	18 (9.1)
Non-ETP-ALL	171 (86.4)
TII	51 (25.8)
TIII	109 (55.1)
TIV	11 (5.6)
T-cell receptor (TCR) chains	
TCRαβ	8 (4.0)
TCRγδ	17 (8.6)
CD4/CD8 expression	
CD4(−)CD8(−)	90 (45.5)
CD4(+)CD8(+)	60 (30.3)
CD4(−)CD8(+)	28 (14.1)
CD4(+)CD8(−)	20 (10.1)
Expression of markers of other lineages	
CD117	54 (27.3)
CD33	39 (19.7)
iCD79a	32 (16.2)
CD13	20 (10.1)
CD56	18 (9.1)
CD19	5 (2.5)
CD64	4 (2.0)
CD15	2 (1.0)

**Table 2:** Expression levels of the studied antigens in diagnostic samples (*n* = 198).

Antigen	Samples with Antigen-Positive Blasts, *n* (%)	Mean Positivity, %	Samples with Different Level of Antigen Expression, *n* (%)		
			Negative (<20%)	Heterogeneous (20–89%)	High (≥90%)
CD2	153 (77.3)	91	45 (22.7)	27 (13.6)	126 (63.6)
iCD3	198 (100.0)	65	0 *	148 (74.7) *	50 (25.3)
sCD3	121 (61.1)	58	77 (38.9)	89 (44.9)	32 (16.2)
CD4	79 (39.9)	59	119 (60.1)	65 (32.8)	14 (7.1)
CD5	180 (90.9)	93	18 (9.1)	28 (14.1)	152 (76.8)
CD7	198 (100.0)	98	0	8 (4.0)	190 (96.0)
CD8	88 (44.4)	70	110 (55.6)	56 (28.3)	32 (16.2)
CD10 **	77 (39.3)	77	119 (60.7)	38 (19.4)	39 (19.9)
CD34	116 (58.6)	67	82 (41.4)	85 (42.9)	31 (15.7)
CD56	18 (9.1)	57	180 (90.9)	14 (7.1)	4 (2.0)
HLA-DR ***	39 (19.8)	62	158 (80.2)	31 (15.7)	8 (4.1)

* The lower threshold for intracellular antigen positivity is 10%. ** The marker was analyzed in 196 samples. *** The marker was analyzed in 197 samples.

**Table 3:** Median fluorescence intensity (MFI) levels of the studied antigens in diagnostic samples.

Antigen	Number of Analyzed Samples, *n* *	MFI of Antigen-Positive Fractions of Leukemic Populations **, Median (Range)	MFI of T Lymphocytes, Median (Range)	MFI Quotient (MFIQ), Median (Range)	*p*
CD2	59	9.15 (0.64–30.93)	13.78 (2.42–30.54)	0.89 (0.03–2.07)	0.0035
iCD3	60	4.87 (0.81–16.47)	7.65 (1.83–15.64)	0.71 (0.11–1.84)	<0.0001
sCD3	101	12.49 (2.96–147.57)	52.54 (22.83–92.69)	0.23 (0.04–1.80)	<0.0001
CD4	74	20.15 (7.76–55.15)	25.24 (10.10–56.15)	0.82 (0.28–2.31)	0.0012
CD5	145	15.92 (2.72–38.82)	27.49 (2.96–74.89)	0.64 (0.07–3.52)	<0.0001
CD7	191	12.22 (0.62–78.54)	6.09 (0.81–21.37)	2.13 (0.22–19.01)	<0.0001
CD8	71	13.75 (2.73–75.22)	32.22 (10.42–83.22)	0.40 (0.10–1.85)	<0.0001
CD45	191	12.67 (0.16–66.88)	43.47 (8.29–112.29)	0.33 (0.00–1.66)	<0.0001
CD48	191	2.71 (0.01–30.52)	30.83 (5.96–63.03)	0.10 (0.00–1.21)	<0.0001
CD56	17	6.12 (1.97–26.09)	31.45 (13.77–66.19) ***	0.25 (0.05–0.76)	<0.0001
CD99	191	127.29 (3.54–649.97)	13.93 (0.93–96.85)	10.13 (0.34–47.05)	<0.0001

* Number of samples with antigen-positive populations (T lymphoblasts, T lymphocytes, and NK cells) that could be properly distinguished and were stained with the same antibody sets and acquired using the same cytometer settings. ** MFI of CD7, CD48, CD45, and CD99 was calculated for the entire leukemic population. *** MFI of NK cells.

**Table 4:** Fluorochrome-conjugated antibodies used in the diagnostic assessment of bone marrow samples from patients with T-ALL. BC, Beckman Coulter; BD, Becton Dickinson; i, intracellular antigen; s, surface antigen.

Fluorochrome	FITC	PE	ECD	PC5.5	PC7	APC	APC-A700/ APC-R700	APC-A750	Pacific Blue/ BV421	Krome Orange/ BV510
Tube 1										
Marker	CD66b	CD19	CD56	CD117	CD33	CD34	CD14	CD45	CD7	sCD3
Clone	80H3	J3-119	N901	104D2D1	D3HL60.251	581	RM052	J33	8H8.1	UCHT1
Company	BC	BC	BC	BC	BC	BC	BC	BC	BC	BC
Tube 2										
Marker	HLA-DR	CD1a	CD7	CD10	CD64	-	CD13	CD45	CD11c	CD11b
Clone	Immu-357	BL6	8H8.1	ALB1	22	-	SJ1D1	J33	BU15	Bearl
Company	BC	BC	BC	BC	BC	-	BC	BC	BC	BC
Tube 3										
Marker	TCRαβ	TCRγδ	CD7	CD8	-	sCD3	CD4	CD45	CD2	-
Clone	IP26A	IMMU510	8H8.1	B9.11	-	UCHT1	13B8.2	J33	RPA-2.10	-
Company	BD	BC	BC	BC	-	BC	BC	BC	BD	-
Tube 4										
Marker	Lysozyme	MPO	CD7	-	iCD22	iCD3	-	CD45	iCD79a	-
Clone	LZ-2	CLB-MPO-1	8H8.1	-	HIB22	UCHT1	-	J33	HM47	-
Company	Thermo Fisher	BC	BC	-	BD	BC	-	BC	BD	-

**Table 5:** MRD-oriented combination used at diagnosis and for subsequent MRD studies. BC, Beckman Coulter; BD, Becton Dickinson.

Fluorochrome	FITC	PE	ECD	PC5.5	PC7	APC	APC-R700	APC-A750	BV421	BV510
MRD tube										
Marker	CD48	CD99	CD7	CD8	CD5	sCD3	CD4	CD45	SYTO41	CD16; CD56
Clone	J4.57	3B2	8H8.1	SK1	BL1a	SK7	RPA-T4	J33	-	3G8; NCAM-16.2
Company	BC	Thermo Fisher	BC	BD	BC	BD	BD	BC	Thermo Fisher	BD

## Data Availability

The original contributions presented in the study are included in the article; further inquiries can be directed to the corresponding author.

## Abbreviations

The following abbreviations are used in this manuscript:

MFC	Multicolor flow cytometry
ALL	Acute lymphoblastic leukemia
MRD	Measurable residual disease
BCP-ALL	B-cell precursor ALL
T-ALL	T-cell ALL
TdT	Terminal deoxynucleotidyl transferase
BM	Bone marrow
ETP-ALL	Early T-cell precursor ALL
EGIL	European Group for the Immunological Characterization of Leukemias
MFI	Median fluorescence intensity
MFIQ	MFI quotient
EOI	End-of-induction
TCR	T-cell receptor

## Appendix A

**Table A1:** Changes in MFI levels of the studied antigens in MRD-positive samples throughout induction therapy.

Antigen	Diagnosis (D0)			Day 15			*p _D0–D15_*	EOI			*p _D15-EOI_*	*p _D0-EOI_*
	Median MFI	Range	N	Median MFI	Range	N		Median MFI	Range	N		
sCD3	12.59	2.96–147.57	93	0.52	0.10–18.54	57	<0.0001	1.69	0.10–15.08	31	0.004	<0.0001
CD4	20.20	7.76–55.15	69	0.78	0.10–31.10	69	<0.0001	1.02	0.10–15.07	36	0.6829	<0.0001
CD5	15.52	2.72–38.82	133	7.90	0.10–32.97	57	<0.0001	11.43	0.28–48.65	31	0.2433	<0.0001
CD7	12.22	0.62–56.94	179	17.09	1.34–39.45	69	0.0222	20.43	3.03–85.93	36	0.1007	0.0009
CD8	13.93	2.73–75.22	66	0.28	0.10–17.49	57	<0.0001	0.62	0.10–14.35	31	0.8565	<0.0001
CD45	12.76	0.16–66.88	179	7.61	0.32–36.55	69	<0.0001	11.49	2.08–48.18	36	0.0195	0.0003
CD48	2.72	0.01–30.52	179	2.67	0.27–38.62	69	0.5170	3.61	0.87–25.56	36	0.2560	0.1361
CD99	127.50	3.54–649.97	179	96.99	10.66–614.65	69	0.3107	69.85	6.66–694.67	36	0.3874	0.2843
